# Healthy living and cancer: evidence from UK Biobank

**DOI:** 10.3332/ecancer.2018.792

**Published:** 2018-01-04

**Authors:** Peter C Elwood, Alex Whitmarsh, John Gallacher, Anthony Bayer, Richard Adams, Luke Heslop, Janet Pickering, Gareth Morgan, Julieta Galante, Sunil Dolwani, Marcus Longley, Zoe E Roberts

**Affiliations:** 1 Division of Population Medicine, Cardiff University, Cardiff CF10 3AT, UK; 2 Dementia Platform, Department of Psychiatry, University of Oxford, Oxford OX1 2JD, UK; 3 Velindre Cancer Centre, Cardiff CF14 2TL, UK; 4 Department of Psychiatry, University of Cambridge, Cambridge CB2 1TN, UK; 5 University of South Wales, Cardiff CF24 2FN, UK; 6 Centre for Medical Education, Cardiff University School of Medicine, Cardiff CF10 3AT, UK

**Keywords:** prospective study, healthy behaviours, cancer incidence

## Abstract

**Context:**

UK Biobank is a prospective study of half a million subjects, almost all aged 40–69 years, identified in 22 centres across the UK during 2006–2010.

**Objective:**

A healthy lifestyle has been described as ‘better than any pill, and no side effects [5]. We therefore examined the relationships between healthy behaviours: low alcohol intake, non-smoking, healthy BMI, physical activity and a healthy diet, and the risk of all cancers, colon, breast and prostate cancers in a large dataset.

**Method:**

Data on lifestyle behaviours were provided by 343,150 subjects, and height and weight were measured at recruitment. 14,285 subjects were diagnosed with cancer during a median of 5.1 years of follow-up.

**Results:**

Compared with subjects who followed none or a single healthy behaviour, a healthy lifestyle based on all five behaviours was associated with a reduction of about one-third in incident cancer (hazard ratio [HR] 0.68; 95% confidence intervals [CI] 0.63–0.74). Colorectal cancer was reduced in subjects following the five behaviours by about one-quarter (HR 0.75; 95% CI 0.58–0.97), and breast cancer by about one-third (HR 0.65; 95% CI 0.52–0.83). The association between a healthy lifestyle and prostate cancer suggested a significant increase in risk, but this can be attributed to bias consequent on inequalities in the uptake of the prostate specific antigen screening test.

**Conclusions:**

Taken together with reported reductions in diabetes, vascular disease and dementia, it is clearly important that every effort is taken to promote healthy lifestyles throughout the population, and it is pointed out that cancer and other screening clinics afford ‘teachable moments’ for the promotion of a healthy lifestyle.

## Introduction

The risk of cancer in the UK population has increased from about 37% for people born in 1930 to around 50% for people born in 1960 [1]. The increase is largely due to the increasing age of the population, but even after standardisation for age, cancer rates have risen over the past ten years by 3% in men and 8% in women [1].

In the UK male population, prostate cancer accounted for 26% and colorectal cancer 13% of all cancers, and in the UK female population, breast cancer accounted for 31% and colorectal cancer 10%. Together, these cancers accounted for over half the malignancies in the total population [2].

There is a large body of literature describing beneficial relationships between a healthy lifestyle, usually defined as non-smoking, a low body weight, regular exercise, a healthy diet and a low alcohol intake and an accompanying risk reduction in a variety of diseases, including cancer. Khaw *et al* [3] described how four healthy behaviours combined predict a four-fold difference in the total mortality in men and women, with an estimated impact equivalent to an additional survival of about fourteen years. Ford *et al* [4] reported that the following of four healthy behaviours at baseline predicted a 78% (95% CI, 72–83%) lower risk of developing a chronic disease (diabetes, heart disease, stroke or cancer) than subjects neglecting the behaviours. Elwood *et al* [5] confirmed all the above and added evidence showing, in a 30-year follow-up, a reduced impairment of cognitive function and a 60% reduction in dementia, attributable to the following of a healthy lifestyle.

In this report, we examine the associations between a healthy lifestyle and incident cancer within the UK Biobank cohort [6].

## Methods

UK Biobank is a prospective study of 502,642 participants aged between 40 and 69 years, identified in 22 centres across the UK during 2006–2010 [6].

Participants were not specifically involved in setting the research questions or the outcome measures, nor were they involved in developing plans for recruitment, design or implementation of the present study. They were consulted over these issues through public meetings and through membership of the independent Ethics and Governance Council. They also remain involved with the study through newsletters, public meetings and consultation. However, neither the funding source nor the participants were asked to advise on the interpretation or writing up of any of the results from the study.

Extensive data were collected from those who agreed to participate, using computerised questionnaires, interviews, physical measurements and biological sample testing. Identification data were obtained to enable long-term follow-up of the subjects through linkage to national datasets on cancers and deaths, and agreement for this was obtained from each participant. Information on incident cancers and deaths was coded according to the 10th revision of the International Classification of Diseases (ICD-10) [7].

At recruitment, participants completed a computerised detailed questionnaire on social issues, lifestyle, smoking, physical activity, diet and alcohol. The answer ‘prefer not to answer’ was available for each question on the behaviours. Height and weight were measured by the clinic staff.

The answers to the questionnaires were used to define the following behaviours.

**Smoking:** Each subject was asked ‘do you smoke tobacco now?’ 429 who preferred ‘not to answer’ were excluded. Participants who responded ‘No’ were judged to fulfil this healthy behaviour. Participants who responded ‘Yes, on most or all days’, or ‘only occasionally’ were judged to fail this behaviour.

**Height and Weight:** Height and weight were measured on 499,537 participants. Those who had a BMI (weight/height2) of under 25 and over 18.5 were coded as ‘healthy’ for this behaviour. Height and weight were measured at recruitment.

**Physical Activity:** Participants were asked to report the number of days each week, and for how long, they did moderate, and/or vigorous physical activity. The criteria used were those defined by the NHS [8], namely 150 minutes or more of moderate activity per week or 75 minutes of vigorous activity per week, or an equivalent combination. 71,365 subjects were excluded for not responding to at least one of the questions.

**Diet:** Participants completed a questionnaire on the computer, which asked questions about the number of portions of cooked or raw vegetables, salad, fresh and dried fruit they consumed each day. No account was taken of other food items. 8,001 subjects were excluded for not responding to at least one of the questions. Subjects who consumed at least five portions of fruit or vegetables each day were defined as ‘healthy’.

**Alcohol:** Participants were asked to report their usual intake of different alcoholic beverages (per week or per month, depending on how frequently they reported drinking alcohol): red wine, white wine, beer/cider, spirits, fortified wine and other drinks. 73,061 subjects who should have answered the monthly questions had missing data and could not be included, because they had answered a previous version of the questionnaire. A further 8,948 participants were excluded for non-response. Healthy behaviour was defined as drinking within the NHS recommended guidelines, namely, 14 or less units per week [9].

**Analyses:** The number of healthy behaviours was modelled as a categorical variable. The reference group was participants with ≤1 healthy behaviour since there were few participants following no healthy behaviour. A test for trend was calculated by treating this variable as a continuous variable. The number of healthy behaviours was also modelled as an ordinal variable in order to estimate the average effect on cancer risk of adopting one additional healthy behaviour.

Cox proportional hazards models were used to analyse the association between lifestyle behaviours and cancer risk. Age was used as the underlying time variable. Participants were followed up from the date of the baseline assessment until the date of any cancer diagnosis, except non-melanoma skin cancer, the date of death or 31 March 2014 (end of follow-up), whichever came first.

All the analyses presented were adjusted for the Townsend deprivation index, an area-based measure of social class [10]. The Townsend scores were calculated for the national census output areas and the subjects were assigned Townsend scores based on the output area that their postcode was located in. Analyses were also adjusted for height.

## Results

Following exclusions, 343,150 (169,715 men and 173,435 women) participants in UK Biobank were included in the present analyses. 26,857 (2%) subjects had to be excluded because of prevalent cancer at baseline, together with 12 subjects with uncertainties in the diagnostic data. Other limitations included the fact that each of the behaviours was used in the analysis as a simple dichotomy, defining ‘healthy’ and ‘unhealthy’ and no attempt was made to use quantitative data.

During a median 5.1 years of follow-up (range 3.5–8.1 years), 14,285 participants (7,944 men and 6,341 women) were diagnosed with cancer, other than non-melanoma skin cancer which was omitted (ICD-10 C00–C97 except C44). 1,651 subjects were diagnosed with colorectal cancer (1,035 men and 617 women) (ICD-10 C18–C20); 2,769 women were diagnosed with breast cancer (ICD-10 C50) and 3,220 men with prostate cancer (ICD-10 C61).

The number of healthy lifestyles followed by participants was inversely associated with the risk of cancer (Table 1). The HR comparing subjects, who followed five healthy behaviours with those who followed none or one healthy behaviour, was 0.68 (95% CI 0.63–0.74). The results for men and women were 0.74 (95% CI 0.66–0.84) and 0.55 (95% CI 0.48–0.64), respectively. Following a healthy lifestyle was also associated with a lower risk of colorectal cancer for men and women (HR 0.75; 95% CI 0.58–0.97) and breast cancer for women (HR 0.65; 95% CI 0.52–0.83). However, in contrast to the protective effect of healthy lifestyle behaviours on all cancers, a healthy lifestyle was associated with an increased risk of prostate cancer for men (HR 1.40; 95% CI 1.16–1.69).

Modelling the number of healthy behaviours as a continuous variable, the HR (95% CI) for all cancers was (HR 0.92 CI 0.91–0.94), i.e., each additional healthy behaviour was independently associated with an average of 8% reduction in risk for all cancers. The HR (95% CI) for colorectal cancer for men and women associated with an extra healthy behaviour was 0.91 (0.87–0.95), and 0.91 (0.87–0.94) for breast cancer for women. For prostate cancer, each additional healthy behaviour increased the risk on average by 6% (HR 1.06; 95% CI 1.03–1.10).

Table 2 shows the separate associations between each healthy behaviour and cancer. Each behaviour was associated with a decreased risk of all cancers, compared to not following the healthy behaviour. Being a non-smoker, compared to current smoking, showed the greatest reduction in risk (HR 0.73; 95% CI 0.69–0.77). A healthy BMI and low alcohol intake were both associated with a lower risk of colorectal cancer and breast cancer, and physical activity also seemed to be associated with a lower risk of breast cancer. For prostate cancer, each healthy behaviour seems to suggest an increased risk of prostate cancer.

## Discussion

With almost half a million subjects, UK Biobank is one of the largest and most detailed studies ever set up [6, 11]. Extensive health and social data were obtained and agreement was obtained from all the subjects for their medical records to be followed indefinitely. The population response to UK Biobank was low [12], and although valid estimates of the prevalence of the behaviours, or the incidence of disease cannot be made with confidence, estimates of association between these can be made with a reasonable degree of confidence [13].

In addition to a low response rate, other limitations include the fact that the definitions of some of the behaviours was limited, and each behaviour was used in the analysis as a simple dichotomy, defining ‘healthy’ and ‘unhealthy’ with no attempt to use quantitative data. Furthermore, ‘smoking’ took no account of ‘ex-smoking’, and ‘diet’ ignored the consumption of foods other than fruit and vegetables.

The data on incident cancer, which are summarised here, have accumulated over a period of only five years yet they present a powerful message on the benefits of a healthy lifestyle. Overall, the analyses give unequivocal evidence of a substantial reduction in cancer associated with the following of a healthy lifestyle. The overall risk of cancer is reduced in the subjects who claimed to be living a healthy lifestyle by about one-third (HR 0.68; 95% CI 0.63–0.74) and all the five healthy behaviours appear to contribute to this overall reduction.

This study and these data therefore enrich the known benefits of a healthy lifestyle and increase the responsibility with which healthcare professionals have to communicate the benefits of healthy behaviours widely, and with conviction [14, 15]. The ability of clinicians to ‘follow the science’, and of patients to exercise ‘rational’ choice is, of course, circumscribed by the contexts – social, cultural, economic and others – in which both live, and usually requires a variety of appropriate ‘nudges’ and support [16].

Our findings concur with those of many other studies. The EPIC research team defined a healthy lifestyle as non-smoking, regular physical activity, moderate alcohol intake and they used as a surrogate for a healthy diet, plasma vitamin C level at baseline. Within the total cohort of one-third of a million subjects in nine European countries, they reported that compared with subjects who followed no healthy behaviour, those who followed all four behaviours had a relative risk of death of 0.25 (95% CI 0.18–0.34), and a relative risk of cancer of 0.27 (95% CI 0.17–0.43) [17]. In the 35-year follow-up of the Caerphilly cohort, healthy living, based on the consistent following of four or five of the behaviours, was associated with a reduction in death (HR 0.40; 95% CI 0.24–0.67), a one-third reduction and a six-year delay in the development of cancer [5].

We also report a 25% reduction in colorectal cancer (HR 0.75; 95% CI 0.58–0.97) and a 35% reduction in breast cancer (HR 0.65; 95% CI 0.52–0.83) (Table 2), and these results are in reasonable conformity with other published data. Thus, Romaguera *et al* [17] reported a reduction of 27% (HR 0.73; 95% CI 0.65–0.81) for colorectal cancer, and about a 15% reduction for breast cancer (HR 0.84 CI 0.78–0.90). In a 24-year follow-up of 81,000 women in the US Nurses’ Health Study, women who followed a healthy lifestyle had an HR for colon cancer of 0.55 (95% CI 0.35–0.87) [18]. Kabat *et al* [19] reported reductions of approximately 35–50% for colon and rectal cancer for men and women and approximately 20% for breast (HR 0.81; 95% CI 0.76–0.87) in a cohort of half a million subjects, and Makarem [20] reported non-significant reductions in colorectal cancer associated with healthy living (HR 0.80; CI 0.64–1.01), and breast cancer (HR 0.87; 95% CI 0.74–1.03).

Our results for the association of healthy living with prostate cancer differ greatly from the results for the other cancers examined, showing a significant increase of about 40% (Table 1). Similar inconsistencies appear to be shown within much of the published literature and although the associations are generally small and non-significant, many suggest a possible increase in risk for prostate, but not other cancers [17, 19, 20]. A few authors, however, report reductions associated with healthy living, but their studies are based on fatal prostate cancer alone [21], or selected subjects with aggressive [22] or advanced cancer [23], or in studies conducted before the prostate specific antibody (PSA) test was introduced [24, 25].

A likely explanation of the increase in prostate cancer in subjects following the healthy behaviours appears to be an effect of screening for prostate cancer. All health screening checks are taken up inequitably, a wide range of beliefs, social, health and lifestyle characteristics being associated with differential service use [26]. The PSA test is likely to be especially sensitive to selection bias from these factors, because it has been developed relatively recently, its value is disputed [27] and it has not been promoted within the UK.

In fact, the issue of possible biases arising from an unequal uptake of the PSA test has been examined within UK Biobank, and it has been shown that a number of socio-demographic, lifestyle and health characteristics, which are predictive for prostate cancer, also define men likely to seek PSA testing [28]. In another study, it has been shown that PSA testing advances a diagnosis of prostate cancer by an average of about 11 years [29]. Selection bias is therefore likely with those who seek PSA testing being those with better lifestyles because they generally care about their health, and PSA testing increasing the likelihood of being diagnosed with a cancer that would otherwise go undetected for on average another 11 years.

It seems likely therefore that the relatively recent availability of PSA testing has distorted what is most probably a reduction in prostate cancer by healthy behaviours, in line with the relationships of the other cancers. While a similar bias from screening is likely to occur in colon and breast cancer, the relevant screening tests were introduced very much earlier and are vigorously promoted in the UK, making selective uptake of the tests much less likely than is the case with the PSA. On the other hand, it could simply be that prostate cancer is less sensitive to lifestyle behaviours, but we are not aware of any data supportive of this explanation.

A similar occurrence of this bias was reported from US Physicians, in whom aspirin-taking was associated with a reduction in prostate cancer (HR 0.59; 95% CI 0.43–0.81) ‘before the PSA era’, while ‘during the PSA era’ the association with aspirin was lost (HR 1.01; 95% CI 0.60–1.69) [24]. A further example was reported by Islami *et al* [24], who found smoking to be associated with an increase in prostate cancer, but only in studies published ‘before the prostate-specific antigen screening era’.

In an evaluation of healthy living, it is essential to keep in mind that there are large reductions in diseases other than cancer, including diabetes [5, 30], vascular disease [5, 31, 32] and dementia [5, 33]. The bottom line on healthy behaviours is that they are of enormous relevance to the overall preservation of health and to survival, and not just to the prevention of cancer or any other single disease.

The magnitude of these benefits of healthy living make a healthy lifestyle extremely attractive, but the responsibility for its maintenance lies ultimately with the subjects themselves [15], and the evidence is that relatively few people follow the healthy behaviours consistently. Thus in 1979, within a cohort of middle-aged men in the UK, only 5% were judged to follow a healthy lifestyle while 40% neglected all or most of the healthy behaviours [5], yet 30 years later these proportions had hardly changed [34].

Perhaps, advice to take up one additional healthy behaviour is the most acceptable message for most subjects. In the present study, each additional healthy behaviour was associated with a reduction of about 8% in cancer, independendent of the effects of the other behaviours. Kirkegaard *et al* [35] reported that for each additional recommendation met, there was a lower risk of colorectal cancer of about 13% (95% CI 4–22%). In another study, a one-point increment in the healthy living score was a 5% (95% CI 3–7%) reduction in total cancer [18]. The authors of the long-term study in Caerphilly reported that had the 2,500 men in their cohort each been urged at baseline to adopt one additional healthy behaviour (any one), and if only half them had complied, then during the following 35 years there would have been a 13% reduction in dementia, a 12% drop in diabetes, 6% less vascular disease and a 5% reduction in total mortality [5].

Elsewhere it is urged that health practitioners should ‘make every contact count’ [36] and screening procedures for cancer provide a most suitable ‘teachable moment’ to urge, encourage, nudge and challenge subjects to consider their lifestyle [16, 37, 38]. In a randomised trial, advice on the healthy behaviours given to subjects within a colorectal screening project led to an increased uptake of a healthy diet and an increase in physical activity [39].

## Conclusions

This study adds powerful evidence to the literature showing large health benefits from healthy behaviours (non-smoking, a low BMI, regular physical activity, a healthy diet and a low alcohol intake). These all contribute to a total reduction of about one-third in cancer risk and possibly a greater reduction in cancer mortality.

The reduction in cancer risk, and cancer mortality, taken together with the reductions in diabetes, vascular disease and dementia, which are not presented in detail here, indicate that healthy living is, as the authors of one paper put it: ‘Better than any pill – *and no side effects’!*[5]

## Conflicts of interest

All the authors declare that they have no conflict of interest.

## Author’s contributions

All of the authors contributed to the conception and design of the study and the interpretation of the data. AW analysed the data, and PCE and GM drafted the manuscript. All the authors gave the final approval for the manuscript to be published.

## Funding

No special funding was sought for the work described in this paper.

## Figures and Tables

**Table 1.** Association between number of healthy behaviours and the risk of all cancers, colorectal cancer, breast cancer and prostate cancer in UK Biobank.

		Number of healthy behaviours							Continuous	
		≤1	2	3	4	5	Trend		Per unit	p-value
**Participants**		18,275	58,184	116,098	108,669	41,894				
**All cancers**										
Overall	Cases	899	2,633	4,979	4,346	1,428				
	HR (95% CI)*	**1.00**	**0.87** (0.81–0.94)	**0.81** (0.75–0.87)	**0.76** (0.71–0.82)	**0.68** (0.63–0.74)	<0.0001		**0.92** (0.91–0.94)	<0.0001
Men	Cases	676	1,861	3,070	1,893	444				
	HR (95% CI)*	**1.00**	**0.90** (0.83–0.99)	**0.86** (0.79–0.94)	**0.80** (0.73–0.88)	**0.74** (0.66–0.84)	<0.0001		**0.94** (0.92–0.96)	<0.0001
Women	Cases	223	772	1,909	2,453	984				
	HR (95% CI)*	**1.00**	**0.73** (0.63–0.85)	**0.64** (0.56–0.74)	**0.63** (0.55–0.72)	**0.55** (0.48–0.64)	<0.0001		**0.90** (0.88–0.93)	<0.0001
**Colorect. ca.**										
Overall	Cases	97	321	639	443	151				
	HR (95% CI)*	**1.00**	**0.99** (0.79–1.25)	**0.99** (0.80–1.23)	**0.78** (0.62–0.97)	**0.75** (0.58–0.97)	0.0001		0.91 (0.87–0.95)	0.0001
Men	Cases	81	252	452	204	46				
	HR (95% CI)*	**1.00**	**1.02** (0.80–1.31)	**1.06** (0.84–1.35)	**0.73** (0.56–0.94)	**0.65** (0.45–0.93)	0.0001		**0.89** (0.84–0.94)	0.0001
Women	Cases	16	69	188	239	105				
	HR (95% CI)*	**1.00**	**0.87** (0.51–1.50)	**0.82** (0.49–1.36)	**0.78** (0.47–1.29)	**0.75** (0.44–1.27)	0.1687		**0.95** (0.87–1.03)	0.1738
**Breast cancer**	Cases	84	356	844	1,063	422				
	HR (95% CI)*	**1.00**	**0.92** (0.73–1.17)	**0.80** (0.64–1.00)	**0.77** (0.62–0.97)	**0.65** (0.52–0.83)	<0.0001		**0.91** (0.87–0.94)	<0.0001
**Prostate Ca**	Cases	195	683	1,251	838	253				
	HR (95% CI)*	**1.00**	**1.12** (0.95–1.31)	**1.17** (1.00–1.36)	**1.18** (1.01–1.38)	**1.40** (1.16–1.69)	0.0010		**1.06** (1.03–1.10)	0.0006

* Results adjusted for Townsend deprivation index and for height.

**Table 2.** Association between each healthy behaviour and the risk of all cancers, colorectal cancer, breast cancer and prostate cancer in UK Biobank.

		All cancers			Colorectal cancer			Breast cancer			Prostate cancer	
Behaviour	Participants	Cases	HR (95% CI)*		Cases	HR (95% CI)*		Cases	HR (95% CI)*		Cases	HR (95% CI)*
**Smoking**												
Current smoker	34,052	1,707	1.00		149	1.00		235	1.00		278	1.00
Non-smoker	309,908	12,578	**0.73** (0.69–0.77)		1,444	**0.92** (0.78–1.09)		2,534	**0.92** (0.81–1.06)		2,942	**1.11** (0.98–1.26)
**BMI**												
≥25 kg/m	225,339	9,907	1.00		1,159	1.00		1,649	1.00		2,376	1.00
<25 >18.5 kg/m	117,811	4,378	**0.93** (0.90–0.96)		434	**0.85** (0.76–0.95)		1,120	**0.90** (0.83–0.97)		844	**1.08** (0.99–1.16)
**Physical activity**												
<150/75 mins mod/vig activity	125,357	5,262	1.00		576	1.00		1,073	1.00		1,097	1.00
≥150/75 mins mod/vig activity	217,793	9,023	**0.93** (0.90–0.96)		1,017	**0.97** (0.88–1.07)		1,696	**0.92** (0.85–1.00)		2,123	**1.07** (0.99–1.15)
**Diet**												
<5 portions fruit/veg per day	64,713	2,662	1.00		278	1.00		381	1.00		647	1.00
≥5 portions fruit/veg per day	278,437	11,623	**0.95** (0.91–0.99)		1,315	**1.01** (0.89–1.15)		2,388	**0.93** (0.84–1.04)		2,573	**1.03** (0.94–1.12)
**Alcohol**												
>14 units per week	141,088	6,363	1.00		795	1.00		824	1.00		1,782	1.00
≤14 units per week	202,062	7,922	**0.95** (0.92–0.98)		798	**0.81** (0.73–0.90)		1,945	**0.87** (0.80–0.95)		1,438	**1.05** (0.98–1.12)

* Results adjusted for Townsend deprivation index and for height and each lifestyle behaviour has been adjusted for the other behaviours.
